# Machine learning accelerated approach to infer nuclear magnetic resonance porosity for a middle eastern carbonate reservoir

**DOI:** 10.1038/s41598-023-30708-7

**Published:** 2023-03-09

**Authors:** Ayyaz Mustafa, Zeeshan Tariq, Mohamed Mahmoud, Abdulazeez Abdulraheem

**Affiliations:** 1 Civil and Environmental Engineering Department, Swanson School of Engineering, University of Pittsburgh, Pittsburgh, PA 15260 USA; 2 Physical Science and Engineering Division, King Abdullah University of Science and Technology (KAUST), Thuwal, 23955 Saudi Arabia; 3 Petroleum Engineering Department, College of Petroleum Engineering and Geosciences, King Fahd University of Petroleum and Minerals (KFUPM), Dhahran, 31261 Saudi Arabia

**Keywords:** Crude oil, Natural gas

## Abstract

Carbonate rocks present a complicated pore system owing to the existence of intra-particle and interparticle porosities. Therefore, characterization of carbonate rocks using petrophysical data is a challenging task. Conventional neutron, sonic, and neutron-density porosities are proven to be less accurate as compared to the NMR porosity. This study aims to predict the NMR porosity by implementing three different machine learning (ML) algorithms using conventional well logs including neutron-porosity, sonic, resistivity, gamma ray, and photoelectric factor. Data, comprising 3500 data points, was acquired from a vast carbonate petroleum reservoir in the Middle East. The input parameters were selected based on their relative importance with respect to output parameter. Three ML techniques such as adaptive neuro-fuzzy inference system (ANFIS), artificial neural network (ANN), and functional network (FN) were implemented for the development of prediction models. The model’s accuracy was evaluated by correlation coefficient (R), root mean square error (RMSE), and average absolute percentage error (AAPE). The results demonstrated that all three prediction models are reliable and consistent exhibiting low errors and high ‘R’ values for both training and testing prediction when related to actual dataset. However, the performance of ANN model was better as compared to other two studied ML techniques based on minimum AAPE and RMSE errors (5.12 and 0.39) and highest R (0.95) for testing and validation outcome. The AAPE and RMSE for the testing and validation results were found to be 5.38 and 0.41 for ANFIS and 6.06 and 0.48 for FN model, respectively. The ANFIS and FN models exhibited ‘R’ 0.937 and 0.942, for testing and validation dataset, respectively. Based on testing and validation results, ANFIS and FN models have been ranked second and third after ANN. Further, optimized ANN and FN models were used to extract explicit correlations to compute the NMR porosity. Hence, this study reveals the successful applications of ML techniques for the accurate prediction of NMR porosity.

## Introduction

Porosity is of vital importance for describing the hydrocarbon reservoirs. It is used for various purposes such as hydrocarbon reserves estimation, describing the reservoir quality, and fluid flow properties within the oil/gas reservoirs. Total porosity is the combination of all types of porosity of the rock formation i.e. effective, isolated porosity and fractures porosity. Generally, it is assumed for the shale free formations (clean formations) and for carbonate formations that effective porosity is equal to total porosity of the formation however it is not true for all cases. Otherwise, correction has to be applied for shale content present in the rock formation as shale presence may lead to the overestimation of formation porosity. There are several techniques of porosity determination in the field and laboratory such like helium porosity in laboratory, mercury injection, nuclear magnetic resonance (NMR), x-ray computed tomography, density logs, sonic logs, neutron porosity. However, among all the techniques, NMR has been found the most reliable and robust technique for the accurate determination of accurate total porosity^1^.

Various researcher investigated and proposed relationships for the porosity estimation in carbonate and other rock formations. Porosity is determined as the ratio between the pore volume (*V*_*p*_) and bulk volume (*V*_*b*_) of the rock formation. Porosity of the clean rock formation could be estimated using Eq. (1) proposed by Wyllie et al.^2^.

1 $$\Phi = \frac{{\Delta T_{\log - } \Delta T_{ma} }}{{\Delta T_{f - } \Delta T_{ma} }}$$

where Δ*T*_log_, Δ*T*_*ma*_, and Δ*T*_*f*_ represent the sonic wave transit time of rock, rock matrix and fluid respectively, in µsec/ft.

Porosity could be estimated from the density as proposed by the Garmard and Poupon^3^.

2 $$\Phi = \frac{{\uprho_{ma - } \rho_{b} }}{{\uprho_{ma - } \rho_{f} }}$$

where ⍴_ma_, ⍴_b_, and ⍴_f_ represent the matrix density, bulk density and fluid density, respectively in g/cc.

The methods for porosity determination such as sonic, density and neutron, present the porosity as a function of lithology. On the contrary, NMR presents the total porosity as a function of hydrogen atoms exist in the fluid and does not depend on the lithology^1^. Although NMR is believed to be a robust and reliable method of estimating the porosity however, factors such as oil viscosity and water salinity could influence the accuracy of NMR porosity^4^.

Timur^5^ introduced the producible porosity for the movable fluid in the rocks that is the ratio between the movable fluid and bulk volume of the rock formation. An empirical correlation was introduced by Timur^6^ for NMR porosity for carbonate rocks on the basis of free fluid index (FFI) data. Porosity was determined using magnetic resonance imaging logs (MRIL) during drilling by the Miller et al.^1^. Another method was introduced by the Chang et al.^7^ to estimate the porosity with great accuracy using combinable magnetic resonance (CMR) tool and conventional logs for complex carbonate formations. High resolution NMR could be obtained using combinable magnetic resonance.

Estimation of total porosity and clay-bound water using NMR logs was also investigated by the Prammer^8^ and found a direct linear relation between transverse relaxation time and water content in clay. The accuracy of NMR porosity was investigated by Georgi et al.^9^ through comparing the NMR porosity with laboratory-based core porosity. Both porosities are found in good harmony with only 5% error between both measurements. Ehigie^10^ compared the different wireline logging tools used for estimating the porosity such like CMR, NMR and MRIL and noticed that MRIL measured porosity was not accurate and reliable in the areas of elliptical breakouts. Dual and triple porosity modal are the primary characteristics of carbonate rock formations which are caused by the presence of fractures, cavities and vugs.

### NMR principle and applications

NMR is an advanced and non-destructive quantitative technique used for the evaluating the pore characteristics and fractures identification^10^. NMR measurements is also able to provide valuable information about the rock properties such as fluid type, fluid state, porosity, pore size distribution. It also provides the sufficient information about the free fluid index and bulk volume irreducible^11^.

NMR measures the porosity by detecting the hydrogen nuclei present in the pore spaces. NMR measure the response of hydrogen nuclei of atoms possessing odd number of neutrons and protons under the influence of applied magnetic field at a certain frequency of resonance. The significantly strong angular momentum of hydrogen nuclei, a strong magnetic moment is developed in the protons due to their magnetic moment, and they start spinning like a magnet bar. The external magnetic field cause the polarization of hydrogen protons and a frequency, known as Larmor frequency, is induced in these protons. This induced frequency is quantitively measured as a quantum property. The NMR measurements also provide the precise estimation of the wettability in reservoir rocks^12^.

The NMR porosity is basically derived from two types of time measurements: longitudinal (T_1_) and transverse (T_2_) relaxation times. The time required by relaxation of the radio frequency pulses to equilibrium energy state is regarded as T_1_ whereas T_2_ is the time required by the secondary magnetic field to loss its phase coherence which is the distinctive feature^13^. Three kinds of relaxation mechanisms control the T_2_ relaxation time including diffusion-induced, bulk fluid mechanism, and surface interaction which are shown in Eq. (3).

3 $$\frac{1}{{{\mathrm{T}}_{2} }} = \frac{1}{{{\mathrm{T}}_{{2{\mathrm{B}}}} }} + \frac{1}{{{\mathrm{T}}_{{2{\mathrm{S}}}} }} + \frac{1}{{{\mathrm{T}}_{{2{\mathrm{D}}}} }}$$

where T_2D_, T_2B_ and T_2S_ are the diffusion induced, bulk, and surface relaxation times, respectively. The diffusion relaxation is resulted from the diffusion of two magnetic fields gradients, the bulk relaxation is related to interaction among the dipole molecules of hydrogen, and the interaction between atoms and solid surfaces cause the surface relaxation^14^.

### Machine learning applications for porosity determination

Various studies have been reported the machine learning applications for the determination of petrophysical properties such as^13,15–18^. The log-based porosity of carbonate reservoirs was successfully predicted using the artificial intelligence (AI) approaches and validated through the core porosity. The performance of reported AI model was very good in terms of accurate prediction with an error of 8% and correlation coefficient of 0.98^13,15^. Hamada and Elshafei^16^ developed an artificial neural network model for the prediction of permeability and porosity of tight gas sand reservoirs by using the resistivity and density logs, and NMR transverse relaxation time (T_2_) as input parameters. The model was validated by comparing the results with core and NMR based porosity and permeability of gas sand reservoirs^11,16^.

The NMR derived free-fluid porosity and vertical permeability were simulated and extrapolated using the two artificial intelligence approaches (fuzzy logic and artificial neural networks). Conventional logs data of two wellbores in Campos Basin, Brazil was used as model inputs. Predicted parameters were optimized through minimizing the errors by average and genetic algorithm approaches. Both techniques performed quite well with high accuracy and low errors^17^. NMR based permeability was calibrated with core permeability by applying the alternate conditional expectations algorithms to improve the accuracy of NMR based permeability estimation. The calibrated NMR permeability exhibited good harmony with the core permeability exhibited 0.87 correlation coefficient. Further, artificial neural networks model was further implemented to synthetically create the permeability and effective porosity in the un-logged zones or interval of sandstone reservoirs^18^.

NMR derived permeability and effective porosity were predicted with good accuracy through the artificial neural networks technique with Cuckoo optimization algorithm. Conventional logs such like resistivity, bulk density, sonic transit time and neutron porosity were used as model inputs to accurately predict the NMR derived porosity and permeability^19^. Although the model accuracy is good with low errors, however, this is a general case as there was no specific rock formation mentioned in this study. NMR log-based parameters were also predicted by Mohaghegh, using ANN for East Texas fields^20^. However, prediction was based on well log data irrespective of rock formation or reservoir. Therefore, the model lacks to provide the accurate solution for specific type of reservoirs such as sandstone or carbonates reservoirs.

NMR log-based permeability was predicted using fuzzy-logic coupled with genetic algorithm optimization technique. Predicted permeability was then used to predict the well producibility of gas bearing siliciclastic reservoir. Model-based predicted permeability exhibited good correlation with experimentally measured core permeability^21^. A comparative study of different machine learning (ML) techniques such as conventional artificial neural networks, fuzzy decision tree, particle swarm optimization, genetic algorithm, imperialist competitive algorithm, and hybrid ML technique, was done to predict the log-based porosity and permeability of the oil reservoir in northern Persian Gulf. Comparison revealed that hybrid machine learning approach performed better than other techniques for predicting the permeability/porosity of oil reservoir in that area. The reliability of the model is reflected by good correlation with laboratory measured permeability/porosity. The model provides the conventional log-based permeability/porosity in general, however, the model is unable to provide information about the porosity and permeability of different geologic formations^22^.

A new machine learning approach was introduced by Ahmadi et al.^22^ for the accurate prediction of permeability and porosity using fuzzy logic (FL-GA) and least square support vector machine (LSSVM) in combination with genetic algorithm. Conventional logs data and NMR parameters, acquired from northern Persian Gulf oil fields, were used as input and output parameters. However, the study provided the general solution without mentioning any geologic formation or type of reservoir^23^.

Although various studies reported the application of different artificial intelligence techniques for the prediction of petrophysical properties. However, the previously reported AI models were developed are either limited to the rock formations other than carbonate rocks (i.e. sandstone) or no rock formation was explicitly mentioned in the studies. One of the previous studies^14^ reported the application of AI for porosity in carbonate rocks using log data. Hence, this study provides the improved and accurate prediction models for porosity in carbonate reservoirs using NMR porosity data. To the best of author’s knowledge, the application of ML for NMR total porosity of the carbonate rock formations has not been reported previously.

As discussed earlier, NMR provides the porosity with great accuracy and reliability as compared to conventional logs however, NMR logging is very expensive and difficult to run, so it may not be available for all the wells. Owing to the importance of NMR porosity, it is deemed necessary to have the NMR porosity for all the wells. So, ML based models for the prediction of the NMR porosity for carbonates reservoirs would be of great significance and enable to obtain the NMR porosity for the wells where NMR logs are not run. In this study, three robust ML approaches including artificial neural networks (ANN), adaptive neuro-fuzzy inference system (ANFIS), and functional networks (FN) were used to develop the prediction models for NMR porosity for carbonates reservoirs. All three models exhibited very good accuracy in terms of high correlation coefficient and low errors for the testing and validation of the model.

### Modelling approaches: ANN, ANFIS and FN

#### Artificial neural network (ANNs)

Artificially neural networks (ANN) is one of the widely used computational intelligence techniques for solving prediction problems, data mining, approximation, and pattern recognition^24,25^. Several types of ANN are being used however, feedforward and backpropagation are more frequently used algorithms of ANN used for training and prediction^26^. ANN uses different learning algorithms, transfer functions and network functions to solve the given prediction problem by delivering the required output prediction.

ANN resolves the given problem by imitating the assemblies and function of the human nervous system. There is an explicit architectural network forms the elements called artificial neurons which are selected on the basis of the nature of the given engineering problem^27^. The artificial neural networks used huge algorithms’ set to establish the connections among the nonlinear variables in order to provide precise, reliable, and consistent prediction results^28^. In general, two kinds of ANN are being employed such as feed-forward neural networks (FFNN) and feed-back neural network (FBNN). FFNN is the simplified and elementary ANN architecture comprised of perceptron of interconnected layers to develop a unidirectional process of transferring information in forward direction. The information of input functions is transferred nonlinearly through the hidden layer activation function in such a way that precise and relevant feature of output is determined^29^. The second type is also popular and extensively used for ANN applications which has identical architectural features as FFNN with additional feature of creating a back loop. The back loop keeps refining the output variable by sending the error information back to adjust the weights until errors are no longer be improved^29^.

The nodes of ANN network interact with each other through a developed pathways with multiple interconnections. Neural network framework may work with one hidden layer of neurons with assigned weights to each node. The input variables are fed as vectors during training phase. The weights are fine-tuned through gradient descent after the computed errors of output are looped back in FBNN. The processing is reiterated until no further improvement in weights and bias is obtained. The weights are updated using gradient descent on the basis of error function derivatives as expressed mathematically in Eq. (4)^29^.

4 $$\Delta {\mathrm{w}}\left( {\mathrm{t}} \right) = \eta *\nabla {\mathrm{E}}*\left( {\mathrm{t}} \right) + \alpha *\Delta {\mathrm{w}}\left( {{\mathrm{t}} - {1}} \right)$$

where E, η, α, Δw, represent the error observed, updated weight, momentum and learning parameters, respectively^28^.

The comparison among different supervised machine learning algorithms revealed that ANN has several advantages over the other algorithms^28^.

1. ANN is capable of dealing with the non-linear and sophisticated relationships between output and input parameters.
2. ANN works efficiently for the problem with high dimensions
3. Complicated functions and non-limiting classification groups containing input and output variables are exhibited by ANN.

The risk of under- and over-fitting is minimized through the strong tuning ability of ANN. In this study, a fully connected feed-forward neural network is used to predict the NMR porosity (Φ_NMR_) using 3594 data points. Training dataset contains seventy percent data points (2516) which were selected randomly to develop the prediction correlation of the model. Testing and validation were done using the rest thirty percent data points (1078).

#### Adaptive neuro-fuzzy inference system (ANFIS)

Adaptive neuro-fuzzy inference system (ANFIS) is an integration of ANNs and fuzzy logic (FL) and capable of learning and reasoning of both techniques (ANNs and FL) simultaneously. ANFIS used the fuzzy logic theory to formulate the mapping of the input and output layers. Neural networks are used to regulate the mapping parameters by leaning functions^30^. Many engineering problems are resolved through the application of adaptive neuro-fuzzy inference system (ANFIS). Accurate prediction models are developed using complicated and nonlinear algorithms of ANFIS which are structured between input and output parameters^31–33^.

#### Functional networks (FN)

The functional network is the modified and straightforward edition of conventional neural networks^34–36^. The FN is a type of supervised machine learning approach mainly utilized for resolving the regression and function approximation complications^37^. The FN model is developed using FN algorithm which undergoes the learning process from the domain and data information. Three major components including input storing unit, muti-layer processing unit, and output storing unit, are involved in the development of FN prediction model. Algorithm determines the modelling parameters itself such as network structure, topology, and neural functions in contrast to the conventional neural networks that work on the pre-defined and fixed number of neurons. Further, the connections between the neurons (biases and weights) are learned and determined during the training process of the neural network modelling^38^. On the other hand, number of neural functions is susceptible and changed during the parametric and structural learning process.

**Figure 1:**
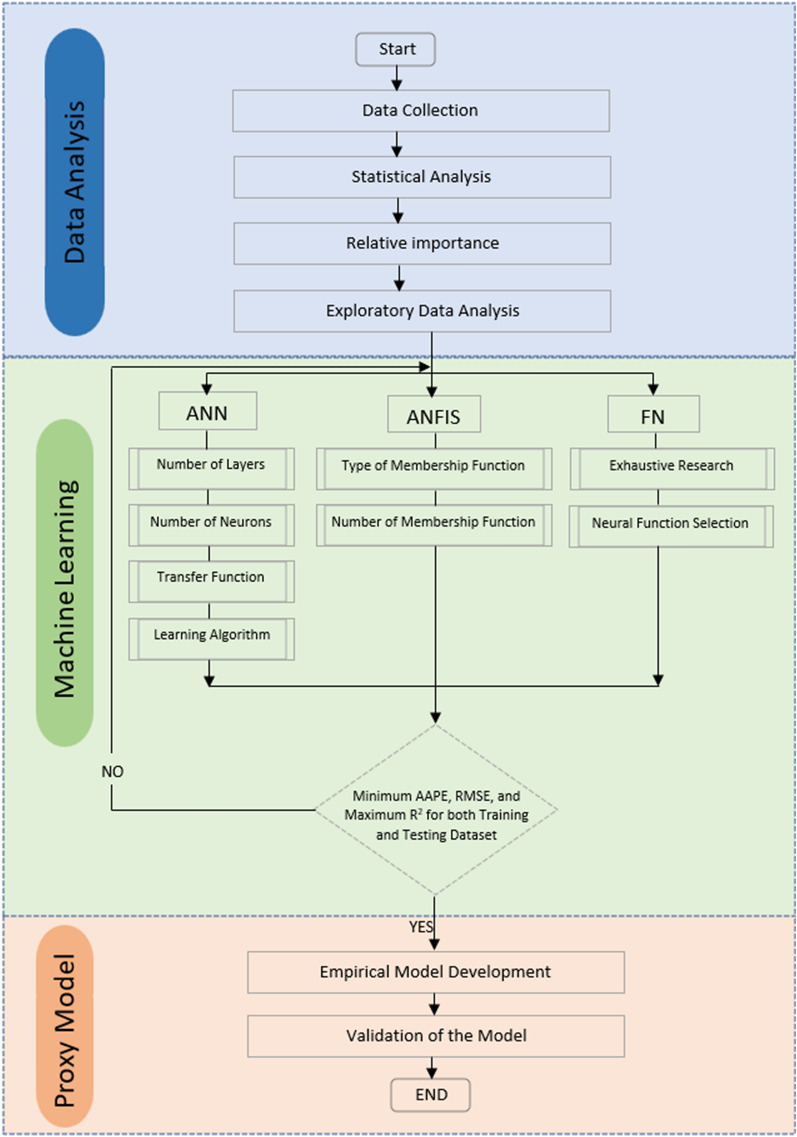
Workflow diagram demonstrating ML modelling approach.

The workflow diagram encompasses the major phases of ML models’ development in this study are illustrated in Fig. 1. Three major components of the study are data analytics, machine learning modelling process and development of models.

### Performance measures

The average absolute percentage error (AAPE), root mean squared error (RMSE), and coefficient of correlation (R) were determined for the predicted values to evaluate the accuracy and reliability of the models. In addition, co Mathematical relations of AAPE and RMSE are shown in Eqs. (5) and (6). Moreover, correlation coefficient (R) was determined as a scale of accuracy for models as well.

5 $${\mathrm{AAPE}} = \left| {\frac{1}{n}\mathop \sum \limits_{i = 1}^{n} \left( {\frac{{\beta_{a} - \beta_{p} }}{{\beta_{a} }}} \right)} \right|$$

6 $${\mathrm{RMSE}} = \sqrt {\frac{{\mathop \sum \nolimits_{i = 1}^{n} [\beta_{a} - \beta_{p} ]^{2} )}}{n}}$$

where ${\beta }_{p}$ and ${\beta }_{a}$ are the predicted and actual values respectively and ‘n’ represents the total number of data points.

The study goals are to provide the convenient, quick, and cost-effective solution for reliable and accurate determination of porosity in complex carbonate reservoirs using conventional log data. The three widely accepted ML approaches including adaptive neuro-fuzzy inference system (ANFIS), artificial neural networks (ANN), and functional network (FN), were implemented to predict the total NMR porosity (Φ_NMR_) through quickly and conveniently and readily available well logs. Machine learning models were developed using conventional wireline logs from carbonate reservoirs. This study offers three ML prediction models for total porosity estimation which is an essential parameter that have strong influence on hydrocarbon storage in the reservoirs.

### Data analysis

**Figure 2:**
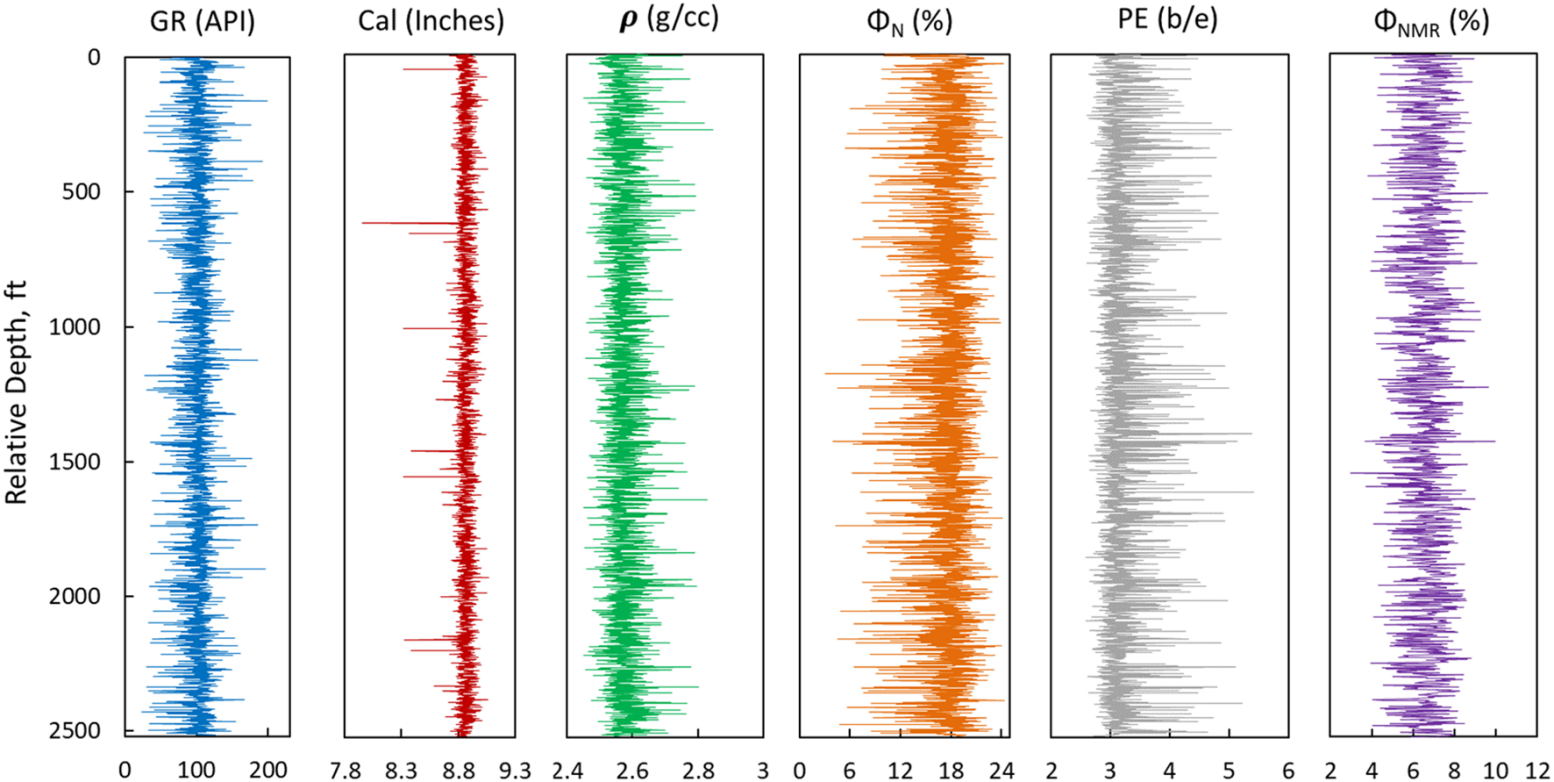
Training dataset used for ML models.

**Figure 3:**
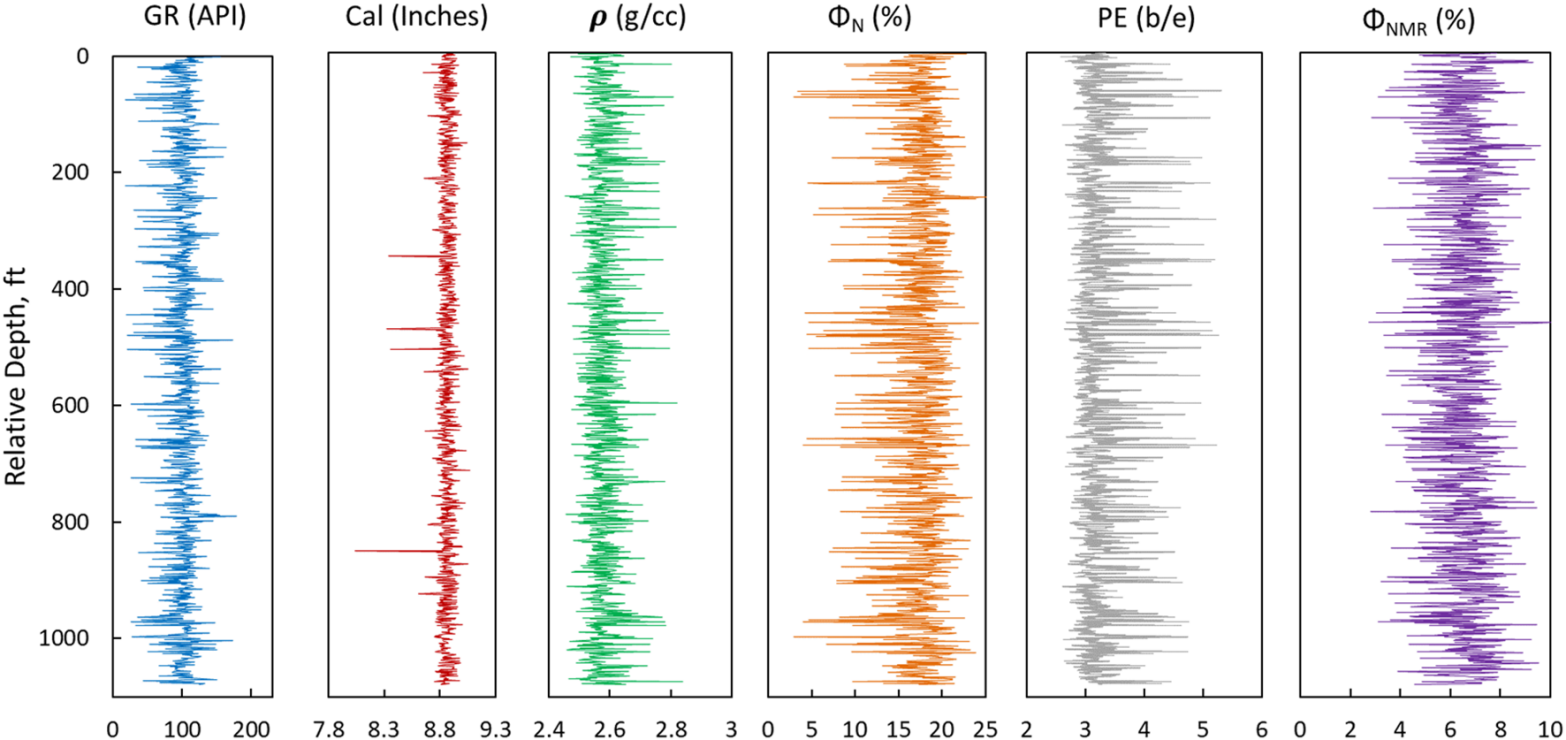
Dataset used for testing of ML models.

Log data, used in this study was acquired from oil wells in carbonate reservoirs containing 3594 data points. Log data such as gamma ray (GR), caliper (Cal), density (ρ), neutron porosity (Φ_N_), and photoelectric factor (PE) were used as input parameters, whereas total porosity (Φ_NMR_) measured from NMR logs is used as output for the ML prediction models. Datasets used for training and testing of ML models are shown in Figs. 2 and 3, respectively.

**Figure 4:**
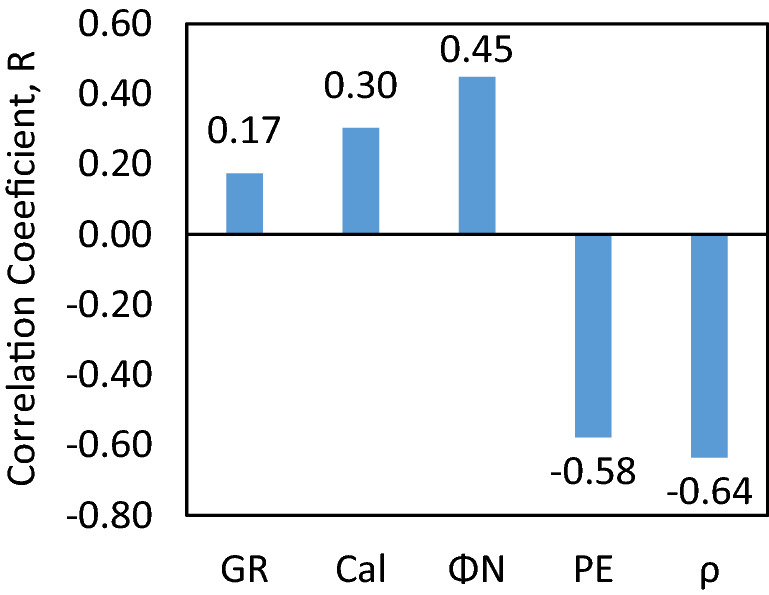
Relative importance of input parameters.

**Figure 5:**
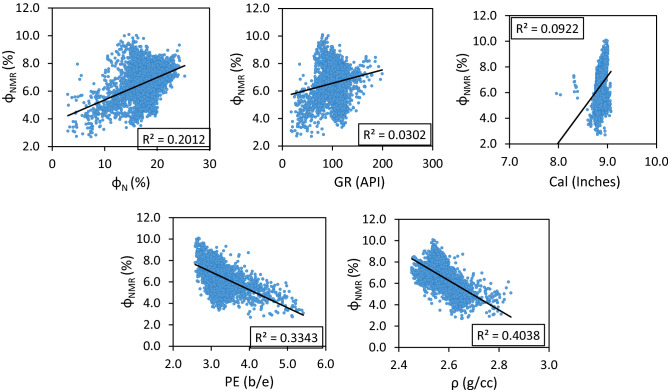
Cross plots of input parameters with Φ_NMR_.

Input parameters exhibited quite good correlation with output parameter NMR porosity (Φ_NMR_) specially density (ρ), neutron porosity (Φ_N_), and photoelectric factor (PE) as reflected by the correlation coefficient (R) (Fig. 4). The strongest correlation was observed for ρ exhibiting R value of -0.64 with Φ_NMR_ and least value was observed for GR case. However, gamma ray has a strong physical relation with rock porosity as it provides the valuable information about the subsurface lithologies, clay volume, other reservoirs characteristics. Due to the geological significance of gamma ray it was included as input parameter. The PE and Φ_N_ also demonstrated good R values (-0.58 and 0.45 respectively) with Φ_NMR_ making them strong candidates for the model input parameters. GR and Cal were also incorporated as the input parameters of models based on their R values (0.17 and 0.30 respectively) and model simulation with different input parameters. Coefficients of correlation and cross-plots between input and output parameters are demonstrated in Figs. 4 and 5, respectively. Furthermore, background knowledge of rock science domain was also used to select the final input variables. Negative correlations were observed for density (ρ), and photoelectric factor (PE) whereas gamma ray (GR), neutron porosity (Φ_N_), and caliper (borehole diameter) have strong positive correlation with NMR porosity (Φ_NMR_). The brief overview of statistical parameters for training dataset (input and output) is shown in Table 1.

**Table 1:** Statistical indicators for training dataset.

Parameters	GR (API)	Calliper (inches)	Neutron porosity (%)	PE (b/e)	Density (g/cc)	NMR porosity (%)
Minimum	24.02	7.96	3.10	2.58	2.45	2.70
Maximum	199.29	9.07	24.30	5.41	2.85	10.02
Mean	101.57	8.86	17.51	3.19	2.58	6.60
Mode	108.24	8.83	17.38	2.97	2.54	6.61
Median	104.33	8.86	18.07	3.10	2.57	6.73
Standard deviation	20.32	0.068	3.14	0.38	0.053	1.15
Kurtosis	2.41	21.03	1.79	5.02	1.70	− 0.20
Skewness	− 0.38	− 2.08	− 1.12	1.98	0.84	− 0.30

Data points of all input parameters are crowded near the mean values exhibiting low variability which is reflected by their low standard deviation. However, higher data variability in GR data is demonstrated by its standard deviation (20.89). GR data points are distributed over a broad range instead of clustering around the mean value. Skewness and kurtosis illustrated the measure of shape of data points. Kurtosis of both training and testing datasets representing almost similar trends. Parameters such as GR, Φ_N_, ρ, and Φ_NMR_, exhibited a platykurtic spread of the data points as reflected by the lower values of kurtosis. The data of these parameters exhibited light tails compared to the normal distribution that represents good quality data with less likelihood of outliers exist in the datasets. Data points of these parameters demonstrated broader and lower peak with lack of extreme values. On the contrary, data points of input parameters such as PE and Cal exhibited a wide distribution with heavier tails. Sharper and higher peaks of data points are observed for these parameters lead to the leptokurtic data distribution. Horizontal axis of histogram is stretched longer, however, most of the data points appear in a skinny (narrow) vertical range. One of the reasons of leptokurtic distribution of Cal and PE is the non-uniform borehole profile representing wash out at various locations in the boreholes.

**Figure 6:**
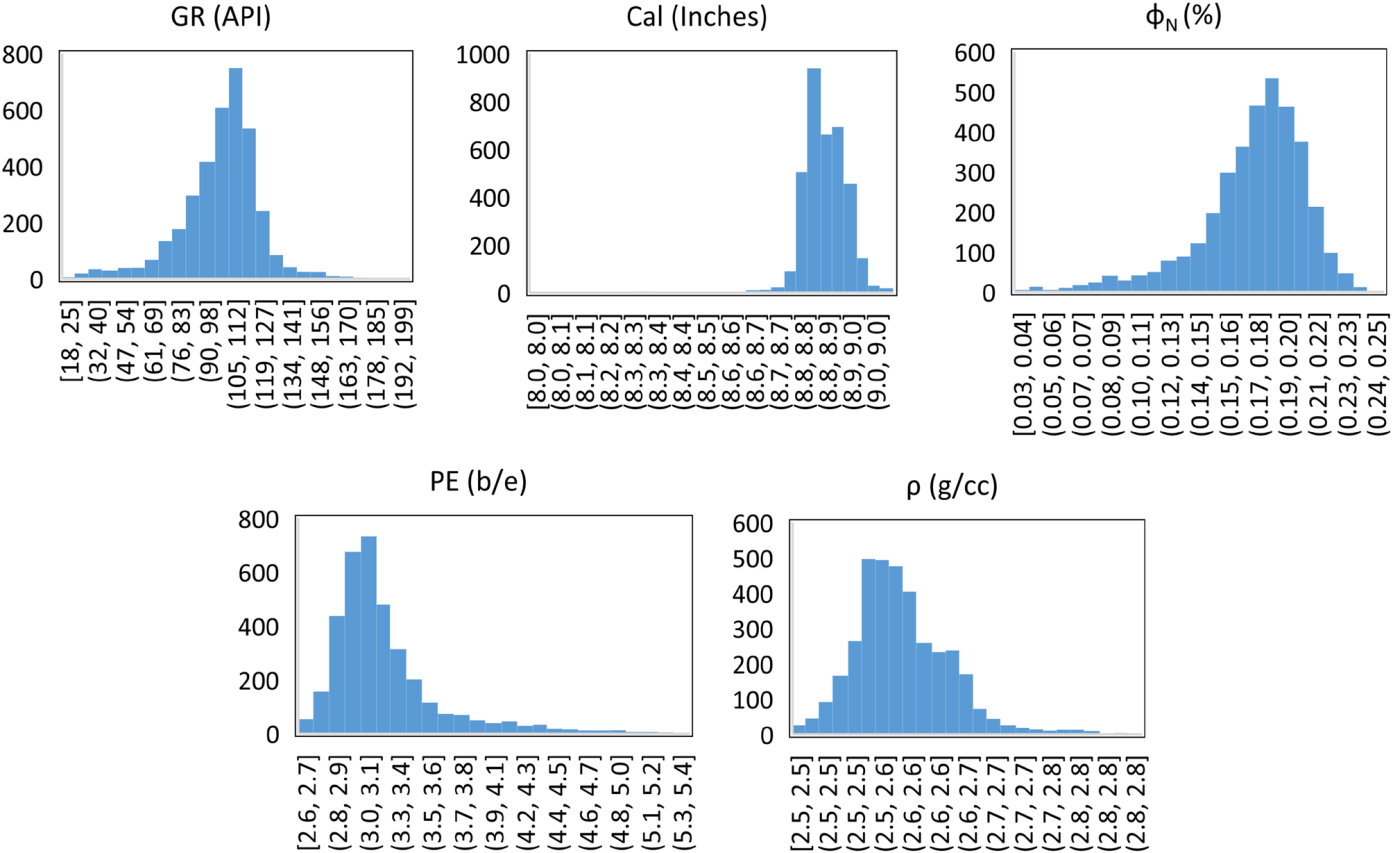
Histogram of input parameters.

Different degree of skewness is reflected by the input and output parameters. Almost, similar behavior in terms of skewness is observed for both the datasets (training and testing) as shown in Tables 1 and 2. Data distribution is fairly symmetrical for GR and Φ_NMR_ parameters with very small skewness. Most of the data points lie around the mean values for these two parameters. Two parameters Φ_N_, and ρ demonstrated a moderate positive and negative skewness respectively. Most of the data points of ρ are less than the mean value exhibiting non-symmetry in the data points whereas; dataset Φ_N_ is dominated by the values higher than the average values. Notably, high skewness was observed for Cal and PE. Cal data contains the values higher than the mean values as reflected by the high negative skewness. On the other hand, high positive skewness is the characteristics of PE dataset indicating that mean value is higher than majority of the data points (Tables 2 and 3). Characteristics of skewness and kurtosis could be observed in the histograms of the input parameters as shown in Fig. 6.

**Table 2:** Statistical indicators for testing dataset.

Parameters	GR (API)	Calliper (inches)	Neutron porosity (%)	PE (b/e)	Density (g/cc)	NMR porosity (%)
Minimum	17.74	8.04	2.96	2.58	2.45	2.73
Maximum	178.51	9.07	25.26	5.31	2.84	10.09
Mean	100.54	8.86	17.27	3.24	2.58	6.52
Mode	NA	8.83	18.31	3.07	2.56	6.82
Median	103.49	8.85	17.98	3.11	2.57	6.68
Standard deviation	22.15	0.07	3.47	0.45	0.06	1.23
Kurtosis	2.29	23.43	2.63	4.86	2.23	0.009
Skewness	− 0.87	− 2.41	− 1.41	2.07	1.11	− 0.39

**Table 3:** Biases and weights connecting the input, hidden, and output layers of ANN Model.

Weights connecting input layer and hidden layer (w_pq_)					
Hidden layer neurons (q)	Input layer neurons (p)				
	1	2	3	4	5
1	− 0.42	1.22	− 1.54	2.27	− 0.07
2	− 3.34	− 0.66	1.87	2.05	− 4.60
3	2.21	− 0.08	1.38	2.72	− 2.79
4	0.66	− 0.34	1.39	0.96	1.33
5	2.68	2.41	1.81	2.38	− 1.42
6	0.37	0.95	1.39	1.32	− 0.98
7	− 2.83	1.58	− 1.51	0.12	0.39
8	− 1.14	− 2.19	1.11	1.77	1.15
9	0.32	0.95	0.31	− 0.48	− 0.12
10	− 1.60	1.54	− 1.37	0.53	2.54
11	− 0.12	− 1.09	0.24	− 0.93	− 1.07
12	1.20	0.03	2.77	0.45	0.42
13	− 0.22	− 2.60	− 0.29	− 0.28	0.60

Bias values for hidden layer neurons			Weights between hidden layer and output layer (w_qr_)		
Hidden layer neurons (q)	Bias (b_q_)		Neurons for hidden layers		Output neuron
1	0.27		1		− 2.16
2	0.57		2		− 0.42
3	− 0.06		3		− 0.98
4	0.65		4		− 0.19
5	0.80		5		− 0.39
6	− 1.64		6		− 0.04
7	0.41		7		− 0.83
8	0.52		8		0.98
9	1.57		9		− 0.62
10	0.26		10		− 1.34
11	0.77		11		0.54
12	0.37		12		1.89
13	− 0.64		13		− 2.73
	Bias values for output layer neuron (b_r_)				
	Output layer neuron		Bias value (b_r_)		
	1		− 0.35

### Models optimization and hyperparameters tuning

#### ANN model

**Figure 7:**
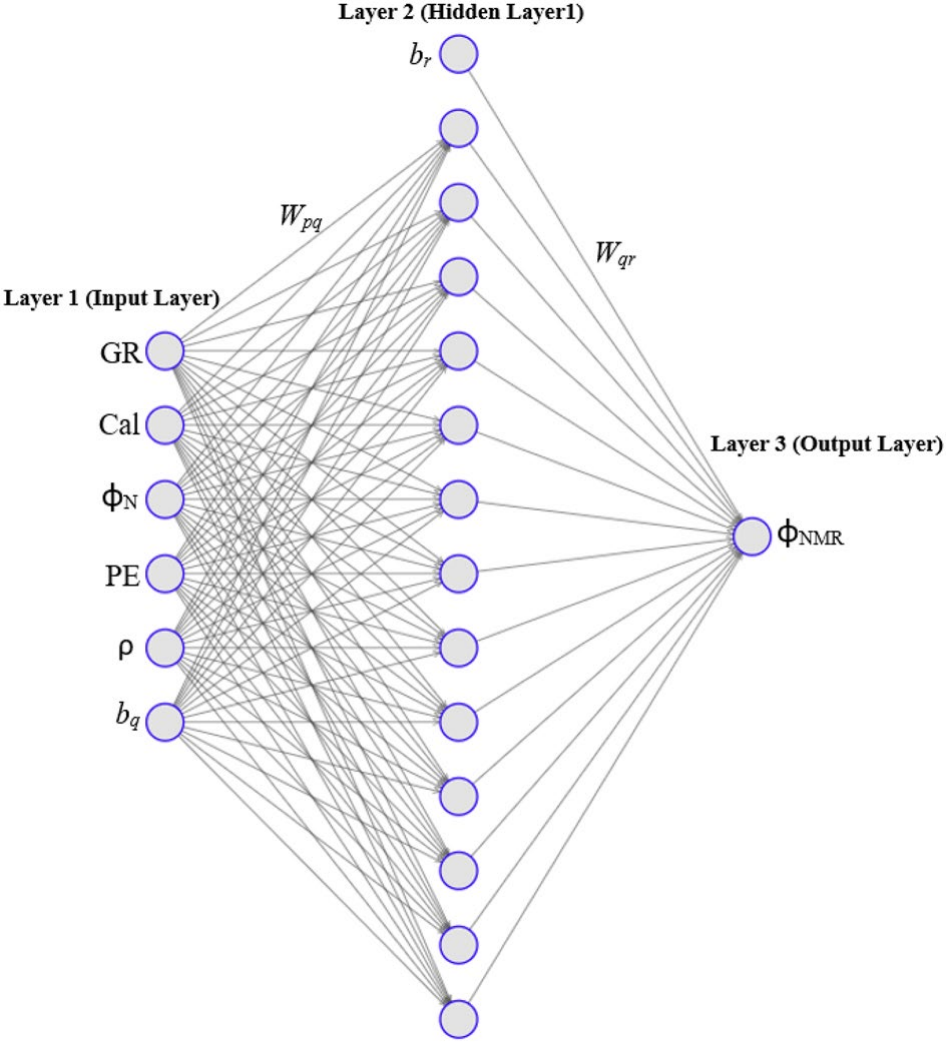
Neuron structure showing the ANN model topology.

**Figure 8:**
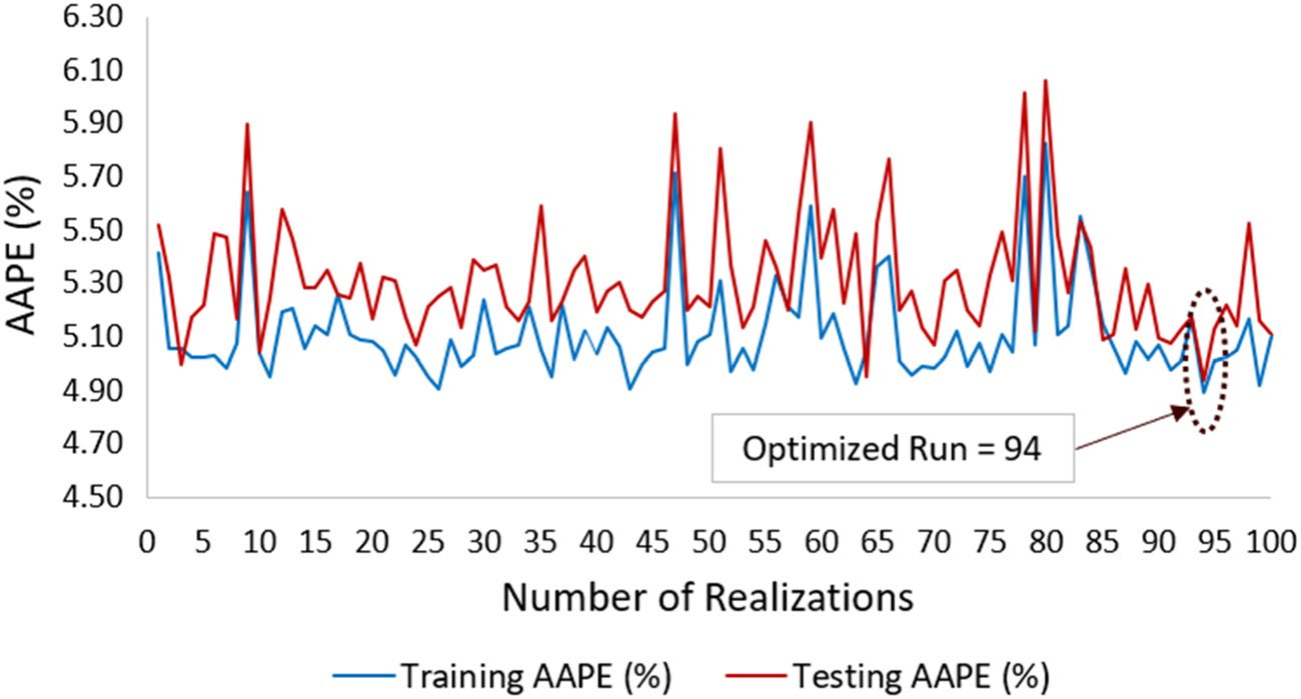
Testing and training AAPE at optimized neurons for 100 realizations.

Artificial neural networks program (ANN) was run at different selected parameters such as number of hidden layers’ neurons, input parameters, and number of realizations in order to optimize the prediction model by minimizing the errors and improving the ‘R’. The number of folds were used for cross-validation. Tangent sigmoidal was selected as an activation function between input and hidden layer and pure linear activation function between hidden and output layer. The number of hidden layers were selected by hit and trial method and Levenberg–Marquardt was used as a learning function. After the optimization of hidden layers in the model, model was run for 100 realizations in order to capture the non-distinctiveness in the dataset. The AAPE errors of ‘training’ and ‘testing and validation’ of ANNs model were compared as shown in Fig. 7. After optimization of the model at different number of realizations and hidden layers neurons, the optimized results were obtained at 13 neurons and 94 realizations exhibiting least errors. Topology of ANN model is shown in Fig. 8. Table 3, shows the biases and weights of the input, hidden, and output layers of the AAN model.the mathematical form of the model is as follows.

7 $$\left( {\Phi_{NMR} } \right)_{N} = \mathop \sum \limits_{q = 1}^{{H_{n} }} W_{qr} n_{hq} + b_{r}$$

8 $$n_{hq} = f \left(\mathop \sum \limits_{p = 1}^{{N_{p} }} W_{pq} i_{p} + b_{q}\right)$$

9 $$n_{hq} = f\left( {(W_{1q} (GR)_{N} + W_{2q} \left( {Cal} \right)_{N} + W_{3q} (\Phi_{N} )_{N} + W_{4q} \left( \rho \right)_{N} + W_{5q} \left( {{\mathrm{PE}}} \right)_{N} + b_{q} } \right)$$

10 $${\mathrm{f}}\left( {\mathrm{x}} \right) = \frac{2}{{1 + e^{ - 2x} }} - 1 = {\mathrm{tanh}}\left( {\mathrm{x}} \right)\;\left( {{\mathrm{Hyperbolic}}\;{\mathrm{Tangent}}\;{\mathrm{Sigmoid}}\;{\mathrm{Transfer}}\;{\mathrm{Function}}} \right)$$

However, normalization was done prior to ANN simulations (using Eq. 11), and de-normalization was done for final output of the model. Equation (17).

11 $$Inputs = \frac{{({\uppsi }_{\max } - {\uppsi }_{\min } )\left( {i - i_{\min } } \right)}}{{(i_{\max } - i_{\min } )}} + {\uppsi }_{\min }$$

where ${\uppsi }_{\min }$ and ${\uppsi }_{\max }$ are – 1 and 1 respectively. For the minimum and maximum input values, please refer to Table 2. The input parameters after normalization, are shown below from Eqs. (12)–(16).

12 $$\left( {GR} \right)_{N} = 0.012 \left( {\left( {GR} \right) - 17.74} \right) - 1$$

13 $$\left( {Cal} \right)_{N} = 1.94 \left( {\left( {Cal) - 8.04} \right)} \right) - 1$$

14 $$\left( {\Phi_{N} } \right)_{N} = 0.09 \left( {\Phi_{N} - 2.96} \right) - 1$$

15 $$\left( \rho \right)_{N} = 5.13 \left( {\rho - 2.45} \right) - 1$$

16 $$\left( {PE} \right)_{N} = 0.732 \left( {PE - 2.58} \right) - 1$$

Equation (17) showing the relationship used for de-normalization of the model output values.

17 $$Output = \frac{{({\uppsi }_{\max } - {\uppsi }_{\min } )\left( {i - i_{\min } } \right)}}{{(i_{\max } - i_{\min } )}} + {\uppsi }_{\min }$$

The values of $i_{\min }$ and $i_{\max }$ used for de-normalization are -1 and 1 respectively. The maximum (${\uppsi }_{\max }$) and minimum (${\uppsi }_{\min }$) values of the output data are 10.09 and 2.07, respectively. For the proposed model, the de-normalization could be shown as shown in Eq. 18.

18 $$\left( {\Phi_{NMR} } \right) = 3.68 \left( {\left( {\Phi_{NMR} } \right)_{N} + 1} \right) - 2.73$$

The weights and biases of input, hidden and output layers of ANN prediction model are mentioned in the Table 3.

#### ANFIS model

The ANFIS model was developed for the prediction of ‘Φ_NMR_’ using the same data points for training and testing. The model was simulated at several different numbers and types of membership functions to achieve the optimized prediction of variable. Though, two membership functions resulted the optimized predicted values after the model was run for different number of membership functions. The optimized results were obtained for gaussian and linear types of membership functions that connect the input, intermediate and output layers.

#### FN model

The FN model was run to establish a prediction correlation for ‘Φ_NMR_’ in addition to ANN and ANFIS techniques. The model simulation was performed using different methods of FN such as Backward-Forward (BF), Forward–Backward (FB), Backward-Elimination (BE), Forward-Selection (FS), and Exhaustive Search (ES) in order to achieve the best prediction results. After the number of runs, ES was selected to for the model development as it demonstrated lowest errors and highest precision in terms of ‘R’. The ES method was executed at linear and non-linear types of functions. The best results with lowest errors and highest ‘R’ were obtained with ES and non-linear types of FN.

Generally, the optimal neural function is obtained through the parametric learning process using a known group of Fourier, polynomial, and trigonometric functions. The study generated a exclusive structure of FN model for ‘Φ_NMR_’ prediction model. Arrangement of estimated neural functions present a focused parameter which is to be predicted. A set of input parameters including GR, Cal, ${\Phi }_{N}$, $\rho$, and PE were regarded as neural functions. The following Eq. (19) showing the parametric learning of this study.

19 $${\Phi }_{NMR} = {\text{ g}}\left( {{\text{GR}},{\text{ Cal}},{ }\Phi_{N} ,{ }\rho ,{\text{ PE}}} \right) = \mathop \sum \limits_{i = 1}^{n} {\upsigma }_{i} {\text{g}}_{i} \left( {{\text{GR}},{\text{ Cal}},{ }\Phi_{N} ,{ }\rho ,{\text{ PE}}} \right)$$

where N, n, σ, represent the size of data set, size of network, and coefficients of neural functions, respectively. The neural functions are determined during the training of the data set. For this study, polynomial group is selected which is denoted by ${1},x,x^{{2}} , \ldots ,x^{h}$ where degree of polynomial is denoted by ‘*h*’.

The architectural design of the functional network consists of five input parameters and five neural functions. Polynomial function (degree, h = 2) is used to acquire these neural functions. The mathematical relation for the ‘Φ_NMR_’ prediction models is illustrated in Eq. (20).

20 $${\Phi }_{NMR} = \mathop \sum \limits_{i = 1}^{k} \omega_{i} \left( {GR} \right)^{{\sigma_{1} }} \left( {Cal} \right)^{{\sigma_{2} }} \Phi_{N}^{{\sigma_{3} }} \rho^{{\sigma_{4} }} \left( {PE} \right)^{{\sigma_{5} }}$$

where number of neural functions and corresponding neural functions are denoted by p and σ (σ_1_, σ_2_, σ_3_, σ_4_, σ_5_) respectively. The numerical values of neural functions and coefficients are for the ‘Φ_NMR_’ model are mentioned in the Table 4.

**Table 4:** Neural functions and respective coefficients.

*P*	Neural functions					Coefficients, ⍵
	${\upsigma }_{1}$	${\upsigma }_{2}$	${\upsigma }_{3}$	${\upsigma }_{4}$	${\upsigma }_{5}$	
1	0	0	0	0	0	19,350.3
2	0	1	0	0	0	− 9858.8
3	0	0	1	0	0	43.5
4	0	0	0	1	0	− 19.8
5	0	0	0	0	1	11,018.8
6	2	0	0	0	0	− 0.0006
7	0	2	0	0	0	1118.7
8	0	0	2	0	0	− 232.5
9	0	0	0	2	0	5.1
10	0	0	0	0	2	− 4199.4
11	3	0	0	0	0	0.000003
12	0	3	0	0	0	− 42.3
13	0	0	3	0	0	575.4
14	0	0	0	3	0	− 0.4
15	0	0	0	0	3	532.6

Likewise other two models, a total of 3594 data points, acquired from different wells, were used for the FN prediction model. Out of total data points, seventy percent (70%) were used for building the model while rest of the 30% were utilized for the evaluation of model’s consistency and reliability.

## Results and discussions

Three ML models were developed to predict NMR total porosity (Φ_NMR_) in carbonate reservoirs using artificial neural network (ANN), adaptive neuro-fuzzy inference system (ANFIS), and functional network (FN) techniques. For all the prediction models, training of the prediction model was executed using seventy percent of the dataset (seen dataset) and testing and validation of the model were done using thirty percent of the data points (unseen dataset). The datasets acquired from different wells in the middle eastern basins. All three ML techniques were run using different parameters, hyper-parameters, and training algorithms to optimize the prediction results.

The ANN model exhibited the AAPE of 5.12, 5.0, and 5.04 for the unseen (testing and validation), seen (training) and overall datapoints respectively indicating high accuracy of the predicted values. The ‘R’ of the predicted values was found to be 0.95, 0.0.94, and 0.944 for unseen (testing and validation), seen (training), and overall data points respectively that reflected the reliability and consistency of the model predicted values. Further, model showed the RMSE of testing and training datasets as 0.39 and 0.39, respectively.

Accuracy of the ANFIS model is quite good with AAPE of 5.38, 5.08, and 5.18 for unseen (testing and validation), seen (training), and overall data points, respectively. The RMSE was found to be 0.39 and 0.41 for seen and unseen data points respectively which affirms the robustness of the prediction model. The ‘R’ of the ANFIS model for the unseen and seen data points was 0.937 and 0.942.

The FN model predicted ‘Φ_NMR_’ values exhibited 5.84 and 6.06 AAPE for the training (model building) and testing datasets when compared to actual data set. The RMSE and ‘R’ for the training (seen) data points were determined as 0.47 and 0.942, and for the unseen (testing and validation) data points as 0.48 and 0.942, respectively. The FN model was also found robust and reliable in terms of accuracy however, the error indices are slightly higher as compared to the other two ML techniques i.e. ANN and ANFIS used in this study. The accuracy indices for all three prediction models are shown in Table 5 in terms of AAPE, RMSE, and ‘R’.

**Table 5:** Accuracy indicators for the ANN, FN and ANFIS models.

Parameter	Model type	Data category	Average absolute percentage error (AAPE) %	Root mean squared error (RMSE)	Correlation coefficient (R)
Φ_NMR_	ANN Model	Training	5.0	0.39	0.948
		Testing and validation	5.12	0.39	0.959
	ANFIS Model	Training	5.08	0.39	0.942
		Testing and validation	5.38	0.41	0.938
	FN Model	Training	5.84	0.47	0.943
		Testing and validation	6.06	0.48	0.942

### Comparison between the ANN, ANFIS, and FN models

#### Accuracy indicators

**Figure 9:**
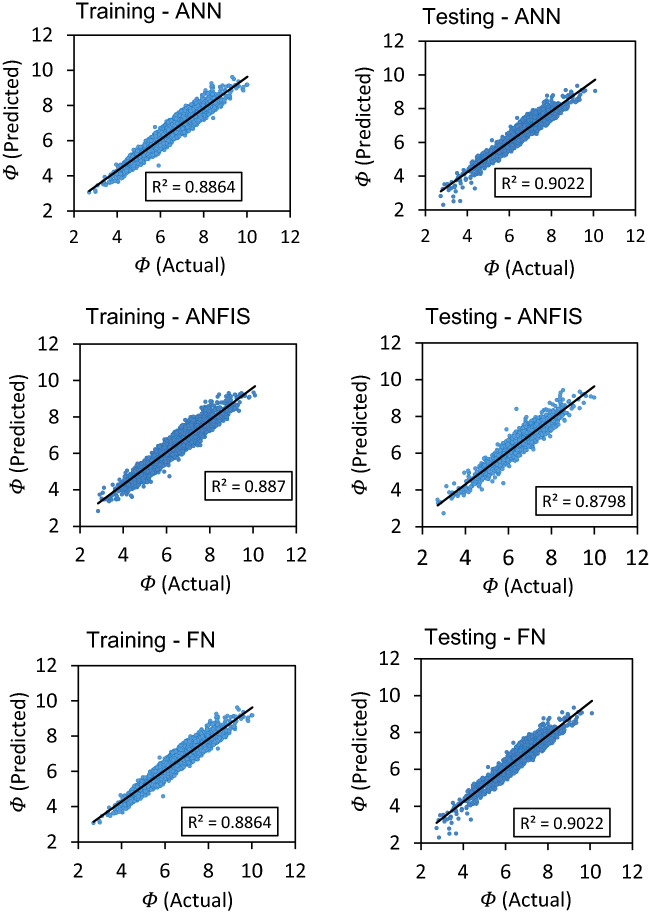
Cross-plots between the predicted and actual Φ_NMR_ values for training and testing dataset of ANN, ANFIS and FN models.

The cross plots between the predicted and actual data points exhibited high coefficient of determination (R^2^) for both seen (training) and unseen (testing and validation) data points as shown in Fig. 9. Among all, the ANN model demonstrated the highest ‘R^2^’ values 0.902 and 0.90 between actual and predicted ‘Φ_NMR_’ for the unseen (testing and validation) and seen (training) data points, respectively. The R^2^ is an excellent indicator for the reliability and accuracy of the predicted values. Comparatively, a slightly lesser ‘R^2^’ were exhibited by the ANFIS model for testing (0.879) and training (0.887) data points (Fig. 9). Likewise, the training and testing datasets of FN model exhibited very good ‘R^2^’ value i.e., 0.889 and 0.888, respectively. However, R^2^ of ANFIS prediction is slightly lesser than ANN and FN models. FN prediction is slightly better than ANFIS model in terms of R^2^. Overall prediction performance of ANN is found to be better than ANFIS and FN. Consequently, all three ML models were found to be robust, steadfast, and consistent as reflected by the R^2^ for the given parameters and their ranges used for model development.

**Figure 10:**
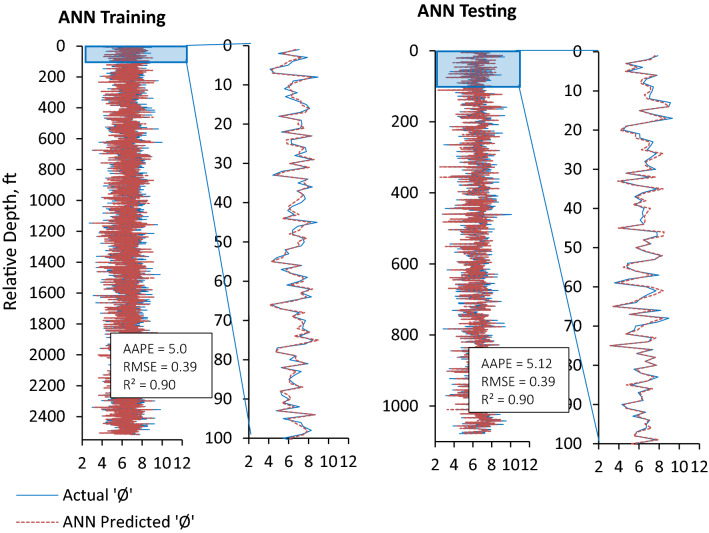
Comparison of training and testing results of ANN model with actual Φ_NMR_.

**Figure 11:**
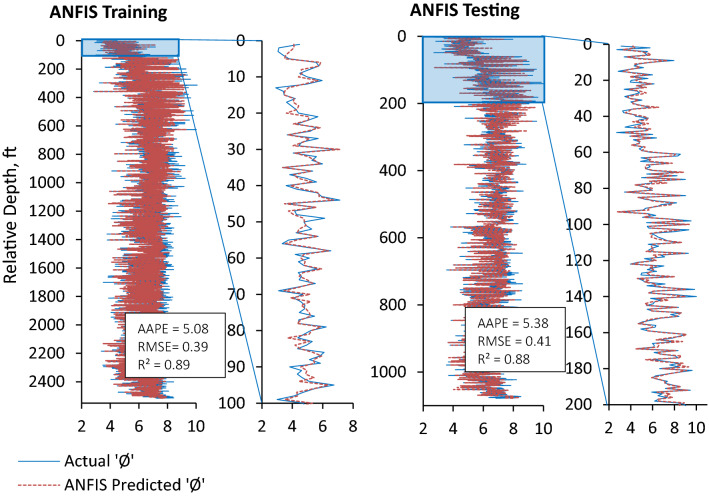
Comparison of training and testing results of ANFIS model with actual Φ_NMR_.

**Figure 12:**
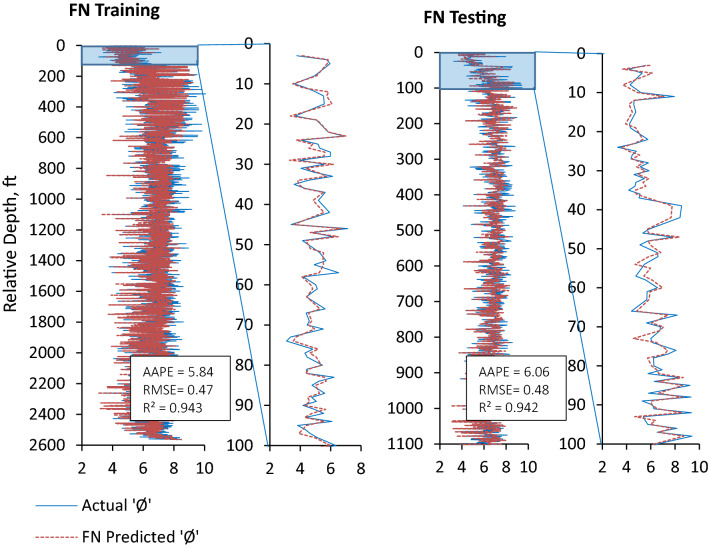
Comparison of training and testing results of FN model with actual Φ_NMR_.

Furthermore, prediction errors (AAPE and RMSE) demonstrated the performance of all three ML prediction models (ANN, ANFIS and FN) to be reliable, consistent, and accurate for the prediction of ‘Φ_NMR_’ with a slight difference in the performance of these models. ANN model outperformed the ANFIS and FN models in terms of lowest AAPE (5.0) and RMSE (0.39) and highest ‘R’ (0.959) for the testing and validation dataset. The ANN model performance for training and testing is shown in Fig. 10. The training and testing performances of ANFIS and FN models could be seen in Figs. 11 and 12 comparing model predicted ‘Φ_NMR_’ and the actual ‘Φ_NMR_’ data. The ANFIS model provides slightly improved prediction compared to FN as demonstrated by prediction errors AAPE (5.08) and RMSE (0.41) for model testing and validation. The AAPE and RMSE were observed to be 6.06 and 0.48 respectively, for the testing and validation of the FN model. The predicted ‘Φ_NMR_’ values are in excellent harmony with the actual data that affirm the applicability and viability of these models for other data sets as well. The performance of ANFIS and FN models is almost the same. ANN model was found to be the most powerful and accurate technique for the prediction of ‘Φ_NMR_’. Therefore, the explicit relation developed for the ANN can be used with confidence to estimate the ‘Φ_NMR_’ using any data set.

All three models were checked thoroughly before the conclusion is drawn. The ANN model was found to be more consistent at both lower and high porosity values with low errors which testify the better performance of ANN model as compared to ANFIS and FN models. however, a marginal difference confirmed that all three models are able to provide the reliable prediction values for NMR porosity.

**Figure 13:**
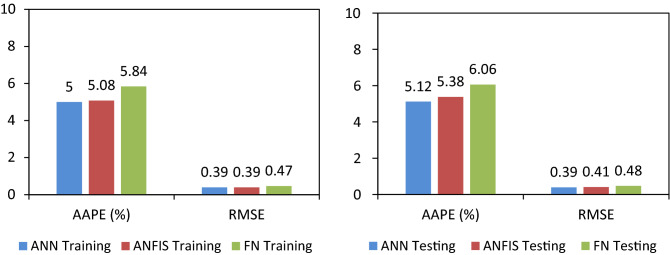
Error comparison of for training and testing dataset for ANN, ANFIS and FN prediction models.

**Figure 14:**
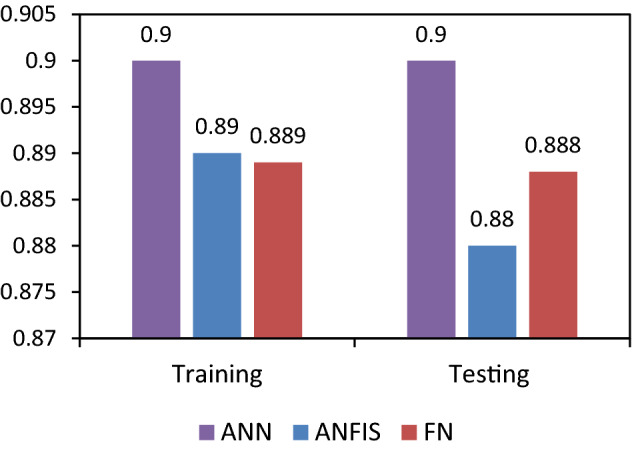
Coefficient of determination (R^2^) comparison between the training and testing datasets of ANN, ANFIS and FN predictions models.

Hence, all thee ML models can be used for the prediction of ‘Φ_NMR_’ using the same parameters and their ranges. The predicted and actual comparison for all three models is shown in Figs. 10, 11, 12 and 13. The errors and ‘R^2^’ for these ML models are depicted in Figs. 13 and 14, respectively.

## Summary and conclusions

In carbonate reservoirs, accurate determination of total porosity of the reservoir has always been a great challenge because of the extremely heterogeneous nature of carbonate pore system. The main motivation of this study is to overcome the challenges for accurate determination of NMR porosity using conventional well logs. Three ML techniques including adaptive neuro-fuzzy inference system, functional networks, and artificial neural networks were employed to build the prediction models. These proposed models were found to be consistent, robust, and viable for precise estimation of NMR-based total porosity in carbonate reservoirs using conveniently available conventional logs. The conclusions of the study are as follows:

1. The proposed ML models would be able to predict the NMR porosity with great accuracy and reliability using readily accessible standard logs such as gamma ray, density, neutron porosity, caliper, and photoelectric factor.
2. The developed models would perform excellent within the range of the input parameters. Further, the ANN-based explicit correlation could be applied confidently to compute the porosity using any other data set that contains the same parameters and ranges. The model can be easily recalibrated to predict the parameters outside the given ranges, once the data is available.
3. All three proposed ML models demonstrated excellent performance and robustness for the prediction of NMR porosity in terms of low AAPE and RMSE errors and high coefficient of correlation.
4. Overall, the performance of ANN prediction model was found to be the best for predicting NMR porosity as compared to the ANFIS and FN models.
5. The proposed ML models exhibited excellent harmony in predicting the porosity for the testing and validation data points.
6. The acquisition of NMR porosity is an expensive and time-consuming job. Thus, developed ML models would provide an intelligent solution to address the problem and make the operations cost-efficient and time-saving.
7. The explicit equation based on ANN trained paradigm would definitely provide a convenient way to the computation of the total porosity without prior knowledge of ML coding language.

## Data Availability

The datasets used and/or analysed during the current study available from the corresponding author on reasonable request.

## References

[1] Miller, M. N., Paltiel, Z., Gillen, M. E., Granot, J. & Bouton, J. C. Spin echo magnetic resonance logging: porosity and free fluid index determination. In *SPE Annual Technical Conference and Exhibition*. Society of Petroleum Engineers. 10.2118/20561-MS (1990).

[2] Wyllie MRJ, Gregory AR, Gardner GHF (1958). An experimental investigation of factors affecting elastic wave velocities in porous media. Geophysics.

[3] Gaymard, R. & Poupon, A. Response of neutron and formation density logs in hydrocarbon-bearing formations. SPWLA (1968).

[4] Elsayed M, Isah A, Hiba M, Hassan A, Al-Garadi K, Mahmoud M, El-Husseiny A, Radwan AE (2022). A review on the applications of nuclear magnetic resonance (NMR) in the oil and gas industry: laboratory and field scale measurements. J. Pet. Exp. Prod. Tech..

[5] Timur, A. Producible porosity and permeability of sandstone investigated through nuclear magnetic resonance principles. SPWLA (1969).

[6] Timur, A. Nuclear magnetic resonance study of carbonate rocks. Society of Petrophysicists and Well-Log Analysts (1972).

[7] Chang, D., Vinegar, D., Morriss, H. J. & Straley, C. Effective porosity, producible fluid and permeability in carbonates from NMR logging. SPWLA (1994)

[8] Prammer, M. G. NMR pore size distributions and permeability at the well site. In *SPE Annual Technical Conference and Exhibition, Society of Petroleum Engineers*. 10.2118/28368-MS (1994).

[9] Georgi, D.T., Shorey, D.S. & Ostroff, G.M. Integration of NMR and conventional log data for improved petrophysical evaluation of Shaly Sands. SPWLA, Oslo (1999).

[10] Ehigie, S. O. NMR-openhole log interpretation: Making the most of NMR data deliverables. In *Presented at SPE Nigeria Annual International Conference and Exhibition*. 10.2118/136971-MS (2010).

[11] Mustafa, A., Mahmoud, M. A. & Abdulraheem, A. A review of pore strcuture characterization of unconventional tight reservoirs. In *SPE Abu Dhabi International Petroleum Exhibition and Conference (ADIPEC), Abu Dhabi*. SPE-197825-MS (2019).

[12] Otchere DA, Mohammad MAA, Ganat TOA, Gholami R, Merican ZMA (2022). A novel empirical and deep ensemble super learning approach in predicting reservoir wettability via well logs. Appl. Sci..

[13] Golsanami N, Sun J, Zhang Z (2016). A review on the applications of the nuclear magnetic resonance (NMR) technology for investigating fractures. J. Appl. Geophys..

[14] Daigle H, Johnson A (2016). Combining mercury intrusion and nuclear magnetic resonance measurements using percolation theory. Transp. Porous Media.

[15] Elkatatny S, Tariq Z, Mahmoud M, Abdulraheem A (2018). New insights into porosity determination using artificial intelligence techniques for carbonates reservoirs. Petroleum.

[16] Hamada, G. M. & Elshafei, M. A. Neural network prediction of porosity and permeability of heterogeneous gas sand reservoirs. In *SPE Technical Symposium and Exhibition*. SPE 126042 (2009).

[17] Carrasquilla AAG, Briones VHT (2019). Simulating porosity and permeability of the nuclear magnetic resonance (NMR) log in carbonate reservoirs of Campos Basin, Southeastern Brazil using conventional logs and artificial intelligence approaches. Braz. J. Geophys..

[18] Al-Ajmi, F. A. & Holditch, S. A. NMR permeability calibration using a non-parametric algorithm and data from a formation in central Arabia. In *SPE Middle East Oil and Gas Show*. SPE 68112 (2001).

[19] Zargar G, Tanha AA, Parizad A, Amouri M, Bagheri H (2020). Reservoir rock properties estimation based on conventional and NMR log data using ANN-Cuckoo: A case study in one of super fields in Iran southwest. Petroleum.

[20] Mohaghegh S (2000). Virtual intelligence applications in petroleum engineering: Part 1—artificial neural networks. J. Pet. Technol..

[21] Malki, H. A. & Baldwin, J. A neuro-fuzzy based oil/gas producibility estimation methods. In *International Joint Conference on Neural Networks*. IEEE (2002).

[22] Ahmadi MA, Chen Z (2018). Comparison of machine learning methods for estimating permeability and porosity of reservoirs via petro-physical logs. Petroleum.

[23] Ahmadi MA, Ahmadi MR, Hosseini SM, Ebadi M (2014). Connectionist model predicts the porosity and permeability of petroleum reservoirs by means of petro-physical logs: Application of artificial intelligence. J. Pet. Sci. Eng..

[24] Mozaffari A, Azad NL (2014). Optimally pruned extreme learning machine with ensemble of regularization techniques and negative correlation penalty applied to automotive engine coldstart hydrocarbon. Neurocomputing.

[25] Dehghani MH, Azam K, Khorasgani FC, Fard ED (2008). Assessment of medical waste management in educational hospitals of Tehran university medical science. Iran. J. Environ. Health Sci. Eng..

[26] Chau KW (2007). Application of a PSO-based neural network in analysis of outcomes of construction claims. Autom. Constr..

[27] Mustafa A, Tariq Z, Mahmoud MA, Radwan AE, Abdulraheem A, Abouelresh MO (2022). Data-driven machine learning approach to predict mineralogy of organic-rich shales: An example from Qusaiba Shale, Rub’al Khali Basin, Saudi Arabia. J. Mar. Pet. Geol..

[28] Otchere DA, Ganat TOA, Gholami R, Ridha S (2021). Application of supervised machine learning paradigms in the prediction of petroleum reservoir properties: Comparative analysis of ANN and SVM models. J. Pet. Sci. Eng..

[29] Saikia P, Baruah RD, Singh SK, Chaudhuri PK (2020). Artificial Neural Networks in the domain of reservoir characterization: A review from shallow to deep models. Comput. Geosci..

[30] MATLAB user guide (2011).

[31] Dogan E, Yiğit MG, Sandalci M, Opan M (2010). Modelling of evaporation from the reservoir of Yuvacik dam using adaptive neuro-fuzzy inference systems. Eng. Appl. Artif. Intell..

[32] Jalalifar H, Mojedifar S, Sahebi AA, Nezamabadi-pour H (2011). Application of the adaptive neuro-fuzzy inference system for prediction of a rock engineering classification system. Comput. Geotech..

[33] Shahrabi MA, Kivi IR, Akbari M, Safiabadi A (2014). Application of adaptive neuro-fuzzy inference system for prediction of minimum miscibility pressure. Int. J. Oil Gas Coal Technol..

[34] Castillo E (1998). Functional networks. Neural Process. Lett..

[35] Castillo E, Cobo A, Gutiérrez JM, Pruneda E (2000). Functional networks: A new network-based methodology. Comput. Civ. Infrastruct. Eng..

[36] Castillo E, Gutiérrez JM, Hadi AS, Lacruz B (2001). Some applications of functional networks in statistics and engineering. Technometrics.

[37] Tariq Z, Abdulraheem A, Mahmoud M, Ahmed A (2018). A rigorous data-driven approach to predict poisson’s ratio of carbonate rocks using a functional network. Petrophysics.

[38] Korany MA, Mahgoub H, Fahmy OT, Maher HM (2012). Application of artificial neural networks for response surface modelling in HPLC method development. J. Adv. Res..

